# Patients with higher vitamin D levels show stronger improvement of self-reported depressive symptoms in psychogeriatric day-care setting

**DOI:** 10.1007/s00702-021-02385-1

**Published:** 2021-07-25

**Authors:** Linda D. Zech, Maike Scherf-Clavel, Christine Daniels, Michael Schwab, Jürgen Deckert, Stefan Unterecker, Alexandra S. Herr

**Affiliations:** Department of Psychiatry, Psychosomatics and Psychotherapy, University Hospital of Würzburg, Margarete-Höppel-Platz 1, 97080 Würzburg, Germany

**Keywords:** Psycho-geriatrics, Vitamin D deficiency, Depression, Anti-depressive treatment

## Abstract

Depression is a common psychiatric disorder among geriatric patients that decreases the quality of life and increases morbidity and mortality. Vitamin D as a neuro-steroid hormone might play a role in the onset and treatment of depression. In the present study, the association between depressive symptoms and vitamin D concentration in serum was evaluated. 140 patients of a psychogeriatric day-care unit were included. The geriatric depression scale (GDS) and the Hamilton depression rating scale (HDRS) were assessed at the beginning and end of treatment, GDS scores additionally 6 weeks after discharge from the day-care unit. Vitamin D levels were measured at the beginning of the treatment, routinely. Patients with levels below 30 µg/L were treated with 1000 IU vitamin D per day. There was no association between the severity of depressive symptoms and the concentration of vitamin D at the beginning of the treatment. Patients with higher vitamin D levels showed a stronger decline of depressive symptoms measured by the GDS during their stay in the day-care unit. We provide evidence that vitamin D serum levels might influence antidepressant therapy response in a geriatric population. Prospective studies are necessary to determine which patients may profit from add-on vitamin D therapy.

## Introduction

Depressive disorders are among the most common mental diseases worldwide (Steel et al. 2014). They are the fifth-leading cause of years lived with disability (GBD 2016 Disease and Injury Incidence and Prevalence Collaborators 2017) and are associated with higher morbidity (Moussavi et al. 2007), mortality (Gilman et al. 2017) and health care costs (Bock et al. 2016). Depressive disorders and symptoms appear at every age and are common in older age, as 10% of those older than 65 are affected (Almeida 2012). Due to the ageing society (Pfötzsch and Rößger 2015), their importance is growing in psycho-geriatrics (Haigh et al. 2018). The anti-depressive treatment in older patients is challenging due to multi-morbidity and poly-medication as well as due to alterations in pharmacokinetics with increasing age (Holvast et al. 2017; Kratz and Diefenbacher 2019). The synthesis of vitamin D starts in the small blood vessels in the skin with the photochemical transversion of the precursor 7-dehydrocholesterol, a metabolite of the cholesterol biosynthesis, to cholecalciferol (25(OH)D_3_) (Fraser 1995, Holick 2007). Bound to the vitamin D-binding protein, cholecalciferol is transported to the liver for the first step of activation, the hydroxylation to 25-hydroxycholecalciferol (25 (OH)D) by the 25-hydroxylase (Bikle 2014; Holick 2007; Haussler et al. 2013), and to the kidney for the second and last step, which is performed by the 25(OH)D-1α-hydroxylase and leads to the formation of 1,25-dihydroxycholecalciferol (1,25 (OH)_2_D), the activated form of vitamin D (Bikle 2014; Holick 2007). Next to its well-known effects on bone health (Wharton and Bishop 2003; Weaver et al. 2016), vitamin D is a neuro-steroid hormone that is discussed to play an important role in the onset and the therapy of depressive symptoms (Anglin et al. 2013; Gowda et al. 2015; Okereke and Singh 2016; Eyles et al. 2005). Receptors for it have been detected in the brain, especially in the prefrontal cortex, the gyrus cinguli, the thalamus and hypothalamus (Eyles et al. 2005). These are regions of the brain that play an important role in the development of depression (Gascon-Barre and Huet 1983; Pardridge et al. 1985). Other facts that lead to the idea of local neurophysiological effects of vitamin D and its potential impact on mood are the presence of the alpha-1-hydroxylase as the key enzyme for the activation of vitamin D in the brain (Eyles et al. 2005) as well as the regulation of neurotransmitters such as serotonin by vitamin D (Dell'Osso et al. 2016; Kraus et al. 2017). In the light of these facts, the scientific interest in vitamin D and its importance in mental health has grown over the past years. Yet, the results are inconsistent due to different kinds of study samples, study designs and different definitions of vitamin D deficiency. Some reported a possible association between vitamin D and depression as well as a positive effect of vitamin D as an antidepressant drug (Anglin et al. 2013; Parker et al. 2017) while others did not find an association between vitamin D deficiency and depressive symptoms (Okereke and Singh 2016; Gowda et al. 2015).

Therefore, the aim of this analysis was to investigate the association between vitamin D status and the severity of depressive symptoms in older patients as well as to evaluate a possible positive effect of vitamin D in anti-depressive therapy.

## Methods

### Patients

Patient data were retrospectively extracted from 183 records of the psychogeriatric day-care unit of the Department of Psychiatry, Psychosomatics and Psychotherapy of the University Hospital of Würzburg between October 2012 and October 2014. This day-care unit is specialized in the treatment of geriatric patients with depression or other psychogeriatric disorders, which are often accompanied by a depressive syndrome. Therefore, as our study was symptom-oriented, all patients were included, regardless of their ICD-10 diagnosis. Only the first stay on the day-care unit was included in patients with multiple stays during this period, so 15 cases had to be excluded. In 141 of these patients, vitamin D concentrations had been measured, routinely in our interdisciplinary geriatric setting. After adjustment for statistical outliers in the vitamin D concentration, 140 patients were included in the analyses. A multimodal therapy program designed and conducted by medical, psychological, and socio-educational experts was applied for treatment. The retrospective analysis of naturalistic clinical data was in accordance with the local ethics committee.

### Measures

#### Depressive symptoms

Depressive symptoms were assessed using the geriatric depression scale (GDS, 30-item version) and the Hamilton depression rating scale (HDRS, 17-item version) at the beginning and end of treatment. Additionally, the GDS was applied 6 weeks after discharge from the clinic. To estimate the change in depressive symptoms during the day-care treatment, the difference of the scores between admission and discharge as well as 6 weeks after discharge from the clinic was calculated.

The geriatric depressive scale is a depression rating scale that in particular is designed to detect depression in older people and contains 30 questions that are answered by the patient himself (Yesavage et al. 1982; Gauggel and Birkner 1999). In contrast, for the HDRS, a standardized interview is conducted by a psychologist who calculates a score by the answers of the patient (Hamilton 1960).

#### Vitamin D levels

Vitamin D (25(OH)D_3_) concentrations in serum routinely are measured at the beginning of treatment at the central laboratory of the University Hospital of Würzburg using an routine assay that is based on chemi-luminescence technology. Patients with levels below 30 µg/L are treated with 1000 IU Vitamin D per day according to the recommendations of the international osteoporosis foundation to reduce the risk of falls and benefit bone health (Bischoff-Ferrari et al. 2009; Chapuy et al. 1992; International Osteoporosis Foundation 2020). The season during the date of the vitamin D measurement was meteorologically classified.

#### Covariates

Furthermore, age, sex, as well as the length of the stay in the day-care unit retrospectively were extracted from the patients’ files.

### Statistical analysis

The mean values (mean) and standard deviations (SD) were calculated for descriptive analyses. As data were not normally distributed (Shapiro–Wilk test), non-parametric tests were performed. Spearman-Rho analysis was used to determine the correlation between two continuous variables. Differences between two groups were analyzed using Mann–Whitney *U* test, differences between more than two groups using Kruskal–Wallis test. For all analyses, *p* ≤ 0.05 was defined as statistically significant.

In every step of the statistical analysis, all patients from whom the corresponding data could be extracted from the files were included. This leads to a changing number of patients in the different statistical calculations but also guarantees the highest possible number of cases in every part of the statistical analysis.

Statistical analyses were performed using IBM SPSS® Statistics, version 25.

## Results

In the patient sample, 48 men and 92 women, aged between 48 and 92 years (mean ± SD = 72.6 ± 7.7 years) were included. Patients were treated in the day-care unit due to different mental diseases. Most patients (74.9%) were suffering from a depressive episode as part of an affective disorder. The minority was diagnosed with a mixed episode of bipolar disorder (4.3%), a manic episode (1.4%), an organic depressive disorder (7.1%), other organically caused mental diseases (1.4%), cognitive dysfunction (3.6%) or Alzheimer’s disease (2.8%), schizoaffective disorder (2.1%), schizophrenia (0.7%) or adjustment disorder (0.7%). As the last-named diseases are often accompanied by depressive symptoms, all patients were included in the analysis. In addition to psychological treatment, the patients were also treated with medication: Focusing on psychiatric medication, 74.3% of the patients were treated with antidepressants, 58.6% of with antipsychotics, 9.3% with benzodiazepines or Z-Substances, 12.9% with anticonvulsive medication, 5.0% with lithium and 3.6% with anti-dementia drugs. Patients spent 36.4 days in mean in the day-care unit. Descriptive data on outcome variables are summarized in Table 1.

**Table 1:** Descriptive data on analysis measures

	*N*	Mean	SD
Vitamin D concentration (µg/L)	140	21.35	10.28
GDS score at admission	127	14	7.1
HDRS score at admission	131	11.4	6.1
Difference of GDS score			
Between admission and discharge	96	− 5.2	6.6
Between admission and 6 weeks after discharge	84	− 4.2	7.8
Difference of the HDRS score between admission and discharge	96	− 6.1	5.3
Days spent in day-care unit	140	36.4	20.8

*GDS* geriatric depression scale, *HDRS* Hamilton depression rating scale, *SD* standard deviation

Vitamin D concentrations in serum were not associated to depressive symptoms established with the GDS (Spearman correlation, *r* = 0.083, *p* = 0.176) or the HDRS (Spearman correlation, *r* = 0.082, *p* = 0.177) at the beginning of the treatment.

**Fig. 1:**
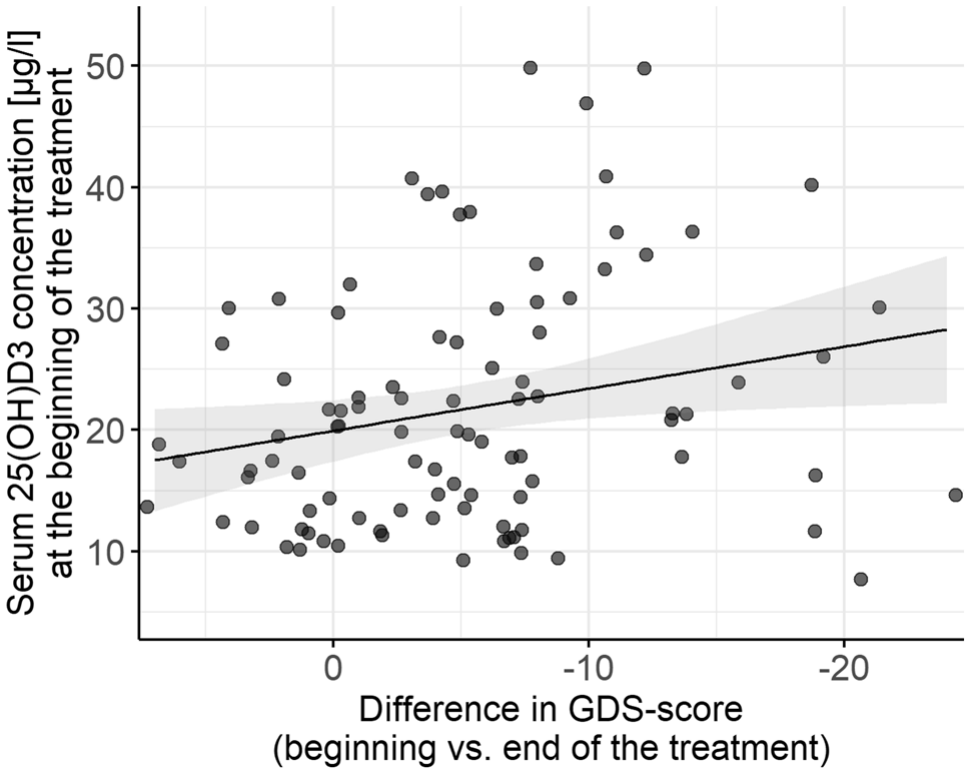
Correlation between vitamin D (25(OH)D_3_) concentration and difference between GDS (Geriatric Depression Scale)—score at beginning and end of treatment (*r* = − 0.243, *p* = 0.008)

However, higher vitamin D concentrations were associated with a higher improvement in depression symptoms from admission to discharge from the clinic, as measured by the GDS (Spearman correlation, *r* = − 0.243, *p* = 0.008, Fig. 1). There was, however, no association between vitamin D levels and the difference of the GDS score between admission and 6 weeks after discharge (Spearman correlation, *r* = − 0.096, *p* = 0.191). Using the HDRS to assess depressive symptoms, the correlation was not significant.

Sex or age was not significantly associated to the vitamin D level. The vitamin D level was found to be significantly correlated with season (Kruskal–Wallis test, *p* = 0.029) with higher levels in fall than in winter (Mann–Whitney *U* test, *p* = 0.023) and spring (Mann–Whitney *U* test, *p* = 0.013).

## Discussion

The present analysis investigated the association between vitamin D status and depressive symptoms and improvement in depressive symptoms in psychogeriatric patients. An association between vitamin D concentration and improvement in depressive symptoms was shown, as measured by a gerontopsychiatric depression rating scale (GDS), but not by the general Hamilton depression rating scale (HDRS).

Our findings suggest that the effect of an antidepressant therapy on self-reported depressive symptoms is greater in patients with higher serum concentrations of vitamin D at the beginning of the therapy. The fact that people with a higher vitamin D level at the beginning of treatment experienced an earlier improvement in depressive symptoms might point to a positive effect of a well-balanced vitamin D status on antidepressant therapy. This is in line with results of a double-blind intervention study which showed that the patient group who received 1500 IU vitamin D and 20 mg fluoxetine showed a significantly higher improvement of depressive symptoms after 8 weeks compared to patients only receiving fluoxetine (Khoraminya et al. 2013). In addition, a study on geriatric patients with major depressive disorder showed similar results — the group who received 300,000 IU vitamin D once showed a higher improvement of their complaints than the placebo group (Zanetidou et al. 2011). Although the study design of these two studies is different from our study, the results suggest a similar effect as a positive influence of vitamin D on depressive symptoms during an anti-depressive treatment is described. Regarding the neurophysiological effects of vitamin D, especially the effect on the release of serotonin (Wang et al. 2005; Jiang et al. 2014), it seems plausible that vitamin D supports the effects of an anti-depressant therapy. The fact that 6 weeks after discharge, this effect was not observed anymore, may be due to the treatment with vitamin D of those patients who had low vitamin D levels at admission, which exerted its beneficial effect on anti-depressant treatment with delay.

In contrast, no association between vitamin D concentration and depressive symptoms at the beginning of the treatment was found. This result is in concordance with a large population-based cross-sectional study (3000 patients, 50 to 70 years old), where no correlation could be found either (Pan et al. 2009). Moreover, one prospective study also reported no association between the serum concentration of vitamin D and depressive symptoms (Toffanello et al. 2014; Jovanova et al. 2017). In contrast, in other cross-sectional studies, the authors detected a significant association (Stewart and Hirani 2010; Lapid et al. 2013). The different results might be based on a different selection of participants as in these studies, the study population was taken from general population and not specifically from a pre-selected group of psychogeriatric patients (Lapid et al. 2013; Stewart and Hirani 2010). Additionally, a different rating scale for depression that was not specifically designed for older patients was used (Lapid et al. 2013) which might explain the different results consistent with our observation. Depressive patients typically tend to spend less time outdoors and to care less about nutrition, especially older people (Wei et al. 2018), which leads to less production of vitamin D in the skin and less intake by food. Therefore, possibly, the vitamin D deficiency is not a cause but a common comorbidity in depressive patients which might be particularly relevant in winter.

We observed higher serum concentrations of vitamin D in fall compared to spring and winter. This result can be explained by the physiological processes to generate vitamin D which are based on the impact of sunlight on the skin. Moreover, the difference between fall and spring/winter can be explained by the fact that the half-life of vitamin D in the human body is 3 months (Preece et al. 1975). This means that the vitamin D storage is filled during the summer and therefore highest in fall, dropping down to the lowest point in winter and spring before it can again be refilled in summer. Other studies are conforming these findings (Klenk et al. 2013; Nanri et al. 2011), where a strong seasonal influence was shown for Vitamin D levels, also specifically for older people (Klenk et al. 2013).

### Limitations

The major limitation was the retrospective character of the analysis. There is an unequal number of men and women, however, this does not affect the results as sex was not associated with the serum concentration of vitamin D. Many of the patients took an antidepressant medication in different combinations, which could have influenced the described effect of vitamin D. To further explore the exact interaction of vitamin D with antidepressants, an interventional study design would be recommended. Somatic diseases and medication were not used as exclusion criteria due to the naturalistic study design. Furthermore, the study sample shows some heterogeneity as patients with different ICD-10 diagnoses were included. Nevertheless, depressive symptoms appear in very many mental diseases, e.g. anxiety disorders or schizophrenia (Tiller 2013; Upthegrove et al. 2017). Our study was symptom- and score-oriented, which is why the ICD-10 criteria were not used to exclude patients from the study.

## Conclusion

In the present analysis, no association between low serum concentrations of vitamin D and depressive symptoms could be found. However, improvement of self-reported depressive symptoms was higher in patients with higher vitamin D concentrations at admission to the clinic. Therefore, it is possible that vitamin D could be an effective supplement to antidepressant therapy. To investigate the potential of vitamin D in antidepressant therapy, further prospective interventional studies are needed to improve the anti-depressant treatment, especially in geriatric people.

## Data Availability

Not applicable.
